# Importance of the knowledge of pathological processes for risk-based inspection in pig slaughterhouses (Study of 2002 to 2016)

**DOI:** 10.5713/ajas.18.0070

**Published:** 2018-04-25

**Authors:** Pedro Sánchez, Francisco J. Pallarés, Miguel A. Gómez, Antonio Bernabé, Serafín Gómez, Juan Seva

**Affiliations:** 1 Departamento de Anatomía y Anatomía Patológica Comparadas, Facultad de Veterinaria, Universidad de Murcia, Espinardo, Murcia 30100, Spain; 2 Veterinary Inspector, Centre de Salut Pública, Conselleria de Sanitat Universal y Salut Pública, Generalitat Valenciana, Orihuela, Alicante 03300, Spain

**Keywords:** Pathological Processes, Pigs, Slaughterhouse, Risk-based Inspections, South-Eastern Spain

## Abstract

**Objective:**

The objective of this work was to determine the prevalence of the pathologies that caused the condemnation of pig carcasses in an area of intensive pig farming and Mediterranean climatology and to evaluate their influence in a risk-based inspection procedure for slaughterhouses.

**Methods:**

A retrospective observational investigation was carried out from 2002 to 2016 into the pathological processes that caused the condemnation of pig carcasses in a slaughterhouse from South-eastern Spain. The seasonal effect on the causes of condemnation carcass was reported. Negative binomial model was used to evaluate the effect of season on the rate of antemortem rejections and post-mortem condemnations. Histopathological examinations were performed to confirm the diagnosis.

**Results:**

The risk of antemortem rejections (0.0564%) was significantly greater in summer (risk ratio [RR] = 1.57). Autumn was associated with higher rate (RR = 1.69) of the total postmortem condemnations (0.1046%). Significantly higher rates of pronounced anaemia (0.0111%) were observed in summer (RR = 3.20). The main causes of anaemia were observed gastroesophageal ulcers and haemorrhagic enteropathies. Significantly highest risk of erysipelas (0.0074%) were observed in autumn (RR = 5.485). About other zoonosis, only eight cases (0.0013%) of carcasses were declared unfit due to tuberculosis lesions. Porcine muscular cysticercosis was not detected. Nevertheless, nonspecific causes such as generalized infections and emaciation represented the half of the condemned carcasses (50.90%).

**Conclusion:**

The pathologies leading to the condemnation of carcasses in this study can be considered representative of the pathologies that affect the pig population from a region with a high intensive production and Mediterranean climatology because this slaughterhouse receives a lot of animals from many farms of different size in a high intensive pig production zone (Mediterranean region). Increased knowledge of environmental factors that may foment the appearance of the diseases is essential for implementing inspection programs based on risk assessment in pig’s slaughterhouses.

## INTRODUCTION

The World Organization for Animal Health (OIE) recognizes that slaughterhouses are a key link for the epidemiological monitoring of zoonoses and all animal diseases [1]. Indeed, many studies on the diseases that affect swine herds are based on pathological processes detected in the slaughterhouse, such as pig respiratory complex [2,3] or the interstitial hepatitis caused by ascariasis [4]. However, there are fewer publications that study the causes for the total condemnation of pig carcasses [5]. In the European Union (EU), the European Food Safety Authority (EFSA) highlights the report of the Nordic Council of Ministers, in which of the 20,581,562 pig carcasses inspected in slaughterhouses during 2005, the highest prevalence of condemnation corresponded to processes related to suppurative inflammations such as abscesses in different parts of the carcass (19%), osteomyelitis (17%), infections caused by tail-biting (14%), and pyaemia (10%) [6]. More recently, Vial and Reist [7] published a study on pigs sacrificed in Swiss slaughterhouses between 2007 and 2012 and found that the most frequent causes for the condemnation of carcasses were abscesses (35% to 36% of total condemnations) and acute processes (21% to 22%). Regarding zoonoses, the prevalence of tuberculosis was less than 0.1%, while erysipelas reached 4.8%. According to these authors the percentage of the condemned carcasses ranged from 0.1% to 0.2% for regular or urgent sacrifice, respectively, but was lower than the percentage registered from 1995 to 2002 in the Czech Republic [8], when 0.57% of a total of 36,028,821 pigs were condemned. A similar prevalence (0.58%) was published for a total of 2,264,428 pigs sacrificed in slaughterhouses in the region of Ontario (Canada) during the years 2001 to 2007 [9]. Osteomyelitis (38.52%), granulomatous lymphadenitis (22.70%) and pleurisy/pneumonia (21.17%) were the main causes of total condemnations carcass (0.24%) of 161,001 pigs sacrificed in two abattoirs of Portugal from March 2006 to July 2012 [5]. In Spain, Peralta [10] mention 0.15%, whereas Martínez et al [11] found a higher proportion (8.5%) of condemned carcasses, although this study focused on pigs with postweanig multysistemic wasting syndrome. Abscesses, cachexia, osteomyelitis and polyarthritis, peritonitis and inflammations of the respiratory system were the main causes for total condemnation described in this report.

For EFSA, the best method to guarantee the effective control of the hazards associated with the consumption of meat is to adopt preventive measures and integrated controls between the farms and the slaughterhouses where the animals are sacrificed [12]. With the purpose of maintaining this sanitary status, the official veterinarian of the slaughterhouse must register and communicate any disease or pathological processes observed mentioning individual animal and the herd they come from. Certain countries of the EU, such as the United Kingdom, Denmark or Finland, have systems for integrated monitoring between pig farms and slaughterhouses, so that the results obtained in the inspections carried out at the slaughterhouse are directly added to the farm data bases. The objective of this monitoring system is to detect changes in the prevalence of the pathologies that affect the animals, so that the welfare and health levels of animals can be improved in the farms of origin [4,13,14]. Application of this system allows farms to be classified according to their health status and correlations to be established between the health conditions and the results of the inspections carried out in slaughterhouses [15].

The environmental factors can condition the animal production systems and affect the appearance of pathological processes detected in the slaughterhouses [16,17]. Although the intolerance of the swine to thermal stress is known, the reports do not agree on whether the sensitivity is greater when the temperature is hotter or colder. In Italy, Vitali et al [18] indicated increase of mortality rates in pigs during transport and lair age at the abattoirs in summer. Opposite, Voslarova et al [19] in Czech Republic or García-Díez and Coelho [5] in Portugal showed greater rates of mortality in pigs during the winter. However, most of these reports do not mention the seasonal impact on the carcass condemnations.

The current specific requirements for the post-mortem in spection of swine sacrificed in slaughterhouses in the EU include a visual inspection and omit systematic operations of palpation and incision [20]. This decision is justified by the fact that the risk associated with microbial cross-contamination is greater than the risk of possible deficiencies in the inspection of the pathological processes by these techniques, such as the detection of tuberculosis by incision of the sub-maxillary lymphatic nodules (LN) or of porcine cysticercosis by incision of the heart lengthwise so as to open the ventricles and cut thorough the interventricular septum [12]. The official veterinarian will decide whether these operations are needed before approving the meat for human consumption based on the epidemiological data of the farm of origin, the information of the food chain of the animals, the conclusions of ante-mortem inspection and the visual detection of post-mortem anomalies [21].

In this study, causes for the condemnation of pig carcasses were determined during a period of fifteen years in a slaughterhouse located in a geographical area of high level pig’s production of Spain and with Mediterranean climate. The objective of this work was to determine the prevalence of the pathologies that lead to the condemnation of pig carcasses and to assess whether there are specific epidemiological characteristics in the pig’s livestock in this region that might influence the established risk-based inspection procedures in the slaughterhouses.

## MATERIALS AND METHODS

### Data source

**Figure 1:**
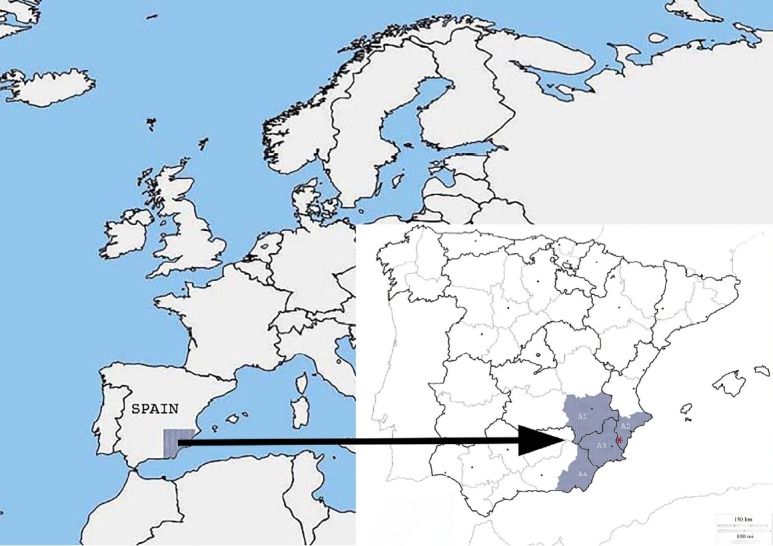
Map of location of slaughterhouse (asterisk) and the study area (South-East of Spain, latitude 38° 5′08″ N and longitude 0°56′49″ W).

The database was collected from the Official Veterinarian Inspection Service records by a Veterinary Inspector at the slaughterhouse under study during fifteen years (2002 through 2016). This slaughterhouse was selected for its representativeness because it receives a lot of animals from many farms of different size from a high intensive pig production zone (Mediterranean area). This abattoir is localized in the South-East of Spain, latitude 38°5′08″N and longitude 0°56′49″W. This area of study comprises a total surface of approximately 40,0761 km^2^ including the border provinces of Albacete (A1), Alicante (A2), Murcia (A3), and Almeria (A4) (Figure 1). All pigs were approximately between 5 to 7 months old and came from farms of this area, so that the animals can be considered representative of the livestock from a wide Mediterranean region. This area is characterized by intensive pig farming activities with a share of sub-desert climate and an annual rainfall of less than 300 L/m^2^. The data regarding the climatological conditions of temperature and rainfall were obtained from the weather station in Elche and Crevillente (Table 1) located in Alicante (Spain). For study purpose, data about number of slaughtered animals by month and year as well as causes of carcass condemnations were obtained. The causes for declaring the pig carcasses unfit for human consumption systematically followed ante- and post-mortem inspection procedures were described in the Spanish legislation [22] between 2002 and 28 May 2006, and after this date in the European Regulation [23]. Since July 2014, a postmortem inspection has been carried out based on the identification of risks for public health considering the European regulations [21].

### Histopathological analysis

In the case of carcasses condemned, which macroscopic inspection enabled an uncertain diagnosis; the presumptive diagnoses were subsequently confirmed in the laboratory. Samples were taken for examination in the Histopathology Laboratory of the Veterinary Faculty of the University of Murcia. For this, one centimeter thick samples were collected from the injured zones and adjacent healthy tissues of affected organs in each pathology process. Likewise, samples of organs without apparent lesion such as lymph nodes were taken in those pathological processes indicated. The samples were collected in polypropylene bottles containing a volume of 10% formalin ten times higher than the sample. The samples were fixed for at least 24 hours and then routinely processed and subjected to an increasing alcohol series. Afterwards, they were embedded in paraffin and 4 μm thick slices were made with the microtome. Finally, they were stained with haematoxylin and eosin (HE). More specific stainings were made in cases of suspected pathologies for which other histopathological stainings are indicated [24], such as periodic acid Shiff (PAS) staining and Grocott staining for fungi, Warthin-Starry (WS) for spirochaetes and Ziehl-Neelsen (ZN) for mycobacteria.

### Descriptive statistic

A retrospective observational study was performed. The total number of ante-mortem rejections and condemned carcasses over the study period by month, season and year were reported. Rates per 100 animals were calculated. The total rate, yearly, seasonal and monthly rates were calculated by dividing the number of animals rejected by the total number of animals entering the abattoir, (condemnations ante mortem) or number of carcass (condemnations post mortem), each year, season or month from 2002 to 2016. These values were then multiplied by 100 to represent the condemnation rate per 100 animals. The category “season” was encoded as follows: January-February-March (winter), April-May-June (spring), July-August-September (summer), and October-November-December (autumn).

### Statistical model

The statistical analysis was performed using SPSS program for Windows version 19.0 (SPSS Inc, Chicago, IL, USA). Statistical associations between ante mortem rejections and causes of post mortem carcass condemnations with the factors season were performed. Interactions between year and season were also examined. Regression models were compared using contrast Chi-square test and the Akaike information criterion. Finally, negative binomial model was used to evaluate the effect of season on the rate of antemortem rejections and post-mortem condemnations. In this analysis only causes of condemnations with more of twenty cases were included. The exposure was the total of pigs entering the abattoir each month and the outcome was the number of animals condemned for each reason of the condemnation. Winter was the season of reference because there are a lower number of postmortem condemnations, which allows a better comparison of the values of the variables. Regression coefficient β (and confidence interval) was transformed into risk ratio (RR) taking the exponent of the coefficient as follows: RR = exp (β). RR is statistically significant when the 95% confidence interval (CI) does not include the unit. A RR>1 means that the incidence rate increases. A RR<1 means that incidence rate is reduced. A p-value <0.05 level was considered as statistically significant.

## RESULTS AND DISCUSSION

### Total condemnation rates

**Figure 2:**
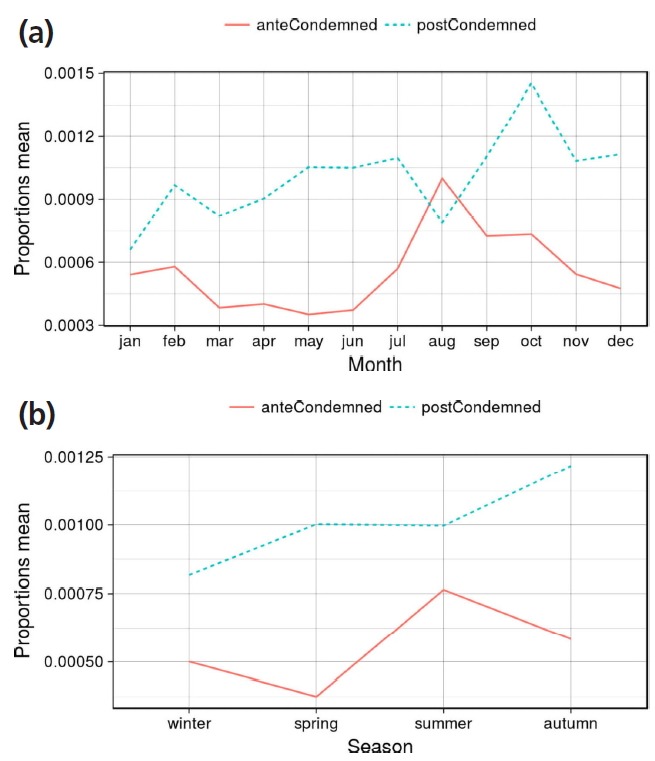
(a) Proportions of antemortem rejections and postmortem carcass condemnation per month of slaughter from 2002–2016. (b) Proportions of antemortem rejections and postmortem carcass condemnation per season of slaughter from 2002–2016.

From a total of 637,002 pigs that arrived at the slaughterhouse, there were 347 (0.0545%) deaths during transportation or deaths in pens, 12 (0.0019%) were condemned at ante mortem inspection and 636,643 sacrificed for human consumption. The number of carcass condemnations after post-mortem inspection was 666, ≈1 condemnation/1,000 animals (0.1046%) (Table 2). The percentage of antemortem and postmortem condemnations by month and season is shown in Figures 2a and 2b. The rainfall (L/m^2^) and temperature (°C) average per season and month of sacrifice is shown in Table 1.

The percentages observed 0.0564% (antemortem rejections) and 0.1046% (carcass condemnations) are similar or lower to those published in recent studies in Europe, rates from 0.049% to 0.15% of finishing pigs dying as result of their transport to slaughter [5,18,19] and rates from 0.1 to 0.24 of carcass condemnations [5,7,10]. We therefore conclude that pig condemnations do not differ significantly from the prevalence of condemnations from swine farms located in other European geographical areas.

### Condemnation causes: unspecific codes

The number of carcasses declared unfit and the percentage of each cause of rejection for the condemned carcasses and the total number of slaughtered animals are shown in Table 2. As can be seen, the main causes for condemnation of carcasses were generalized diseases (227 cases, 0.0357%), which include inflammation in different organs and diseases such as septicaemias, viremias, and pyaemias, or undernourishment (112 cases, 0.0176%). Both represent the half of the condemned carcasses (50.90%). Only 15.16% the condemned carcasses were attributed to acute inflammations located in a single organ as cause of febrile syndrome, especially pleuropneumonia (0.0061%), arthritis (0.0044%) and peritonitis (0.0036%). These results show a lack of specificity in the data concerning to the etiology of pathological processes from carcasses of animals intended for slaughter declared unfit for consumption. This agrees with the findings of several authors and could be attributed to the fact that the etiology of these processes is not usually investigated, except in cases of suspected zoonosis, because it has no influence on the evaluation of the meat [10]. Another reason is the detection of different lesions in several organs of the same carcass [25]. Besides, it is difficult to draw conclusions from the records because the heterogeneity of the classification criteria used [26]. Nevertheless, the registration of anomalies and communication must be improved and standardized. Only if this is done can the nature and intensity of the controls applied by the official veterinarians in pig slaughterhouses be safely based on risk assessment, on the epidemiological data of the farms of origin and by the information relative to the animal food chain.

### Organoleptic alteration by anaemia

The 20% of condemnations were associated with organoleptic alterations. Of special interest in post-mortem inspection was the study of the pathological processes that caused severe anaemia (0.0111%). So, we investigated 71 cases of carcasses declared unfit for human consumption due to severe anaemia (Table 3). Thus, gastroesophageal ulcers were diagnosed in 34 animals, representing 47.9% of the anaemias identified in the sacrificed pigs, and which accounted for 5.10% of the carcasses declared unfit for human consumption. This represents only 0.0053% the total number of sacrificed pigs, whereas the incidence in pig livestock bred on intensive conditions is estimated to be between 6% and 15%, even if only severe cases are considered [27]. If the post-mortem inspection is limited to visual detection, gastroesophageal ulcers can go unnoticed. But since these may be a sign of animal stress, an additional inspection of the gastric mucosa should be performed, at least when anaemia seems to be frequent to identify possible deviations in the welfare of the sacrificed animals [12].

**Figure 3:**
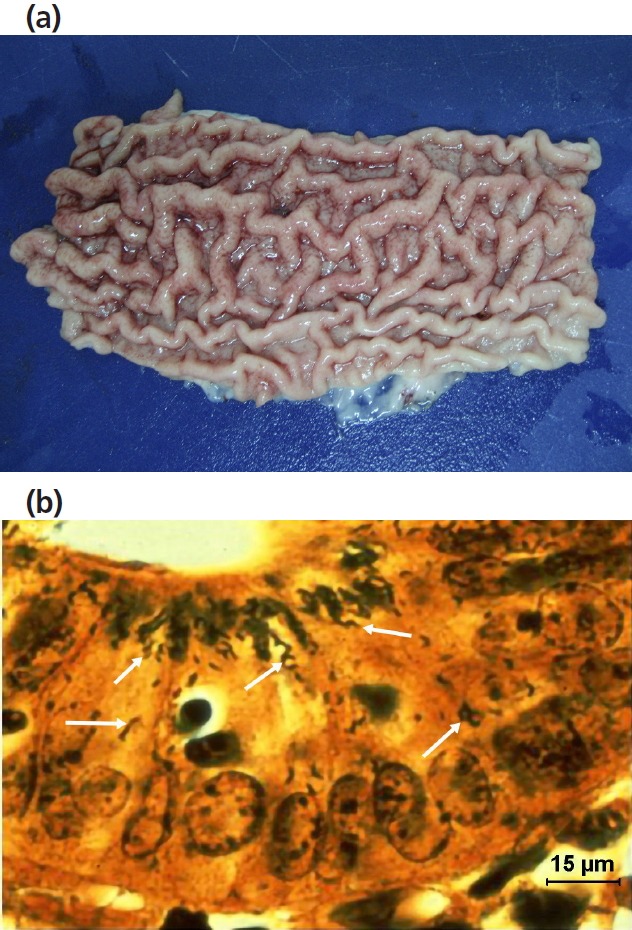
(a) Image of ‘brain circumvolutions’ in the ileum mucosa in a case of anaemia caused by haemorrhagic proliferative enteritis. (b) Microscopic image of haemorrhagic proliferative enteritis (Figure 2a) with *Lawsonia intracellularis* (arrow) within the enterocytes (Warthin-Starry).

**Figure 4:**
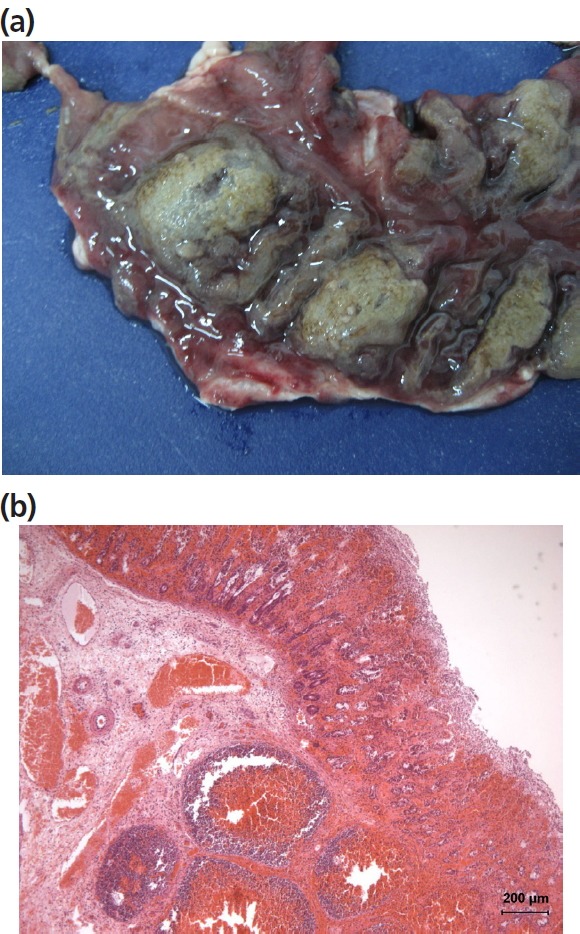
(a) Necrotic areas in the colonic mucosa in a case of pig anaemia caused by haemorrhagic dysentery. (b) Microscopic image of haemorraghic dysentery (Figure 3a) with haemorrhagic inflammation and necrosis on the mucosa (Haematoxylin and eosin).

After additional examinations, 34 cases of haemorrhagic enteritis were confirmed as the cause of anaemia and to have an infectious aetiology. The detailed inspection of the intestinal mucosa in pigs with anaemia allowed us to detect cases of haemorrhagic porcine proliferative enteropathy and of haemorrhagic porcine dysentery. The detection of lesions in the ileum, ‘tile-paved’ images through the serosa and ‘brain-like circumvolutions’ in the mucosa [28] point to proliferative enteritis (Figure 3a). The histopathological lesions of epithelial hyperplasia, glandular adenomatosis, necrosis, haemorrhages and thrombosis of blood vessels and the detection (Figure 3b) of curved intracellular bacteria in epithelial cells (a morphology compatible with *Lawsonia intracellularis*) by WS silver staining were considered sufficient evidence to establish the diagnosis [29]. In the cases of porcine haemorrhagic dysentery, lesions were detected in the large intestine, where the ileocaecal valve was plainly evident; the mucosa showed focal fibrin deposits and necrotic areas (Figure 4a). The histopathological examination revealed lesions of haemorrhagic inflammation, superficial necrosis of the mucosa and the presence of bacteria morphologically compatible with spirochaetes (Figure 4b). The observation of these lesions could show the level of veterinary care on-farm and suspect antibiotic use. Likewise, the influence on the prevalence of salmonella positive swine carcasses could be investigated.

In the case of leucosis, the increased size of the LN and the lardaceous aspect of the lesions upon cutting, allowed a macroscopic diagnosis, except the renal lesions, which may be macroscopically confused with interstitial nephritis lesions [30]. It was necessary to histopathological examination, when infiltration of lymphoblast cells and the absence of signs of inflammation were sufficient to confirm leucosis. The prevalence of leucosis (0.0004%) was lower than that described (0.0015%) in other slaughterhouses [31], probably because we only registered the forms of presentation that led to the total condemnation of the carcasses.

The official veterinarian may declare a carcass unfit for consumption based on a visual inspection of severe organoleptic alterations, without investigating the causes. However, we found that relevant information on the sanitary conditions of origin’s farm should be extracted by performing additional examinations.

### Zoonosis

The two major zoonotic diseases that would involved the application of supplemental incision examinations in postmortem inspection regulated by EU legislation are tuberculosis and swine cysticercosis [23].

In hospitals of this geographical area cases of human neurocysticercosis have been detected, but all correspond to the immigrant population coming from South America [32]. Besides, in the EU the pig population in intensive farming is free of *Taenia solium* [6]. Since porcine muscular cysticercosis caused by *Cysticercus cellulosae* was not detected in any of the 559,529 pigs to which this systematic procedure of inspection was applied until June 2014, there are no epidemiological data indicating that it is necessary to adopt the incision systematic of the heart lengthwise during post-mortem inspection.

The eight cases (0.0013%) of carcasses declared unfit in June 2002 due to tuberculosis lesions corresponded to 85 pigs from a single farm. In another eight pigs, lesions were found in submaxillary or mesenteric LN. In this case it was necessary to perform histopathological examination and ZN staining to confirm the macroscopic diagnosis of tuberculosis. Following growth in selective medium in the National Laboratory of Reference, the *Mycobacterium* involved was identified as *Mycobacterium avium*. However, of the eight cases, one presented organ lesions, in spleen and liver. With the current system of visual inspection only this one case would have been detected, since the rest of the lesions corresponded to LN. Therefore, in cases of granulomatous lymphadenitis, systematic re-inspection of the carcasses from the same batch by means of LN incision is necessary. The prevalence of tuberculosis found in the slaughterhouse is variable and some studies found a higher prevalence of granulomatous lymphadenitis cases (0.06%) [5], *Mycobacterium avium* is the main cause of granulomatous lymphadenitis. This variation in the number of cases could be associated with the geographical region by the different degree of presence of the host and the sanitary measures applied in the farms. In the last fourteen years of the study period no cases of tuberculosis were detected in the pigs sacrificed in the slaughterhouse in question, suggesting that no specific conditions exist on pig farms that would call for the systematically application of submaxillary LN incision.

### Seasonal influence

**Figure 5:**
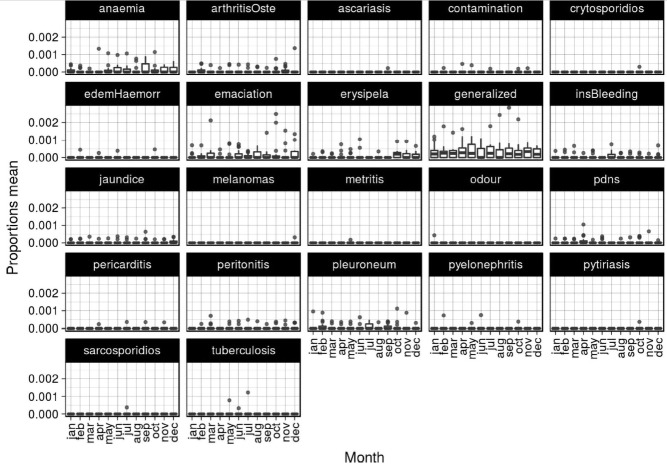
Bloxpot of mean proportion in different condemnation causes per month of slaughter from 2002–2016.

**Figure 6:**
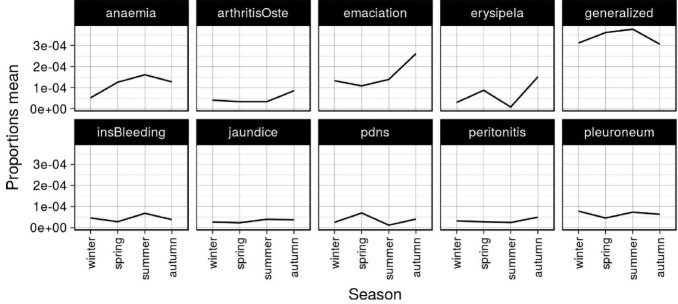
Linear graph of mean proportion in different condemnation causes per season from 2002–2016 (causes with more than twenty cases).

The mean proportion of different condemnation causes by month is presented in Figure 5. The effect of season on the causes involving more than twenty cases of condemnation carcass is reported in Table 4. The mean proportion by season is presented in Table 4 and Figure 6.

The risk of antemortem rejections was significantly greater in summer (0.0765%, RR = 1.57). The highest mortality rates were recorded in August (Figure 6), which was the hottest month. These results can be explained by the high temperatures during the summer (average T^a^ = 26°C) and the mild temperatures during the winter months (average T^a^ = 13°C) compared with other places in Europe. Therefore, in slaughterhouses located in places of similar climatology it would be important increase the monitoring of animal welfare conditions in antemorten inspection during the hottest months of the year.

In contrast to the observations of other authors [5], we founded a statistically significant association within season of year and some causes of carcass condemnations. Autumn was associated with higher rate of the total postmortem condemnations (RR = 1.69, statistically significant). So, autumn was the season with highest risk of generalized, emaciation, erysipelas, arthritis, peritonitis and jaundice.

Significantly higher rates of anemia were observed in summer (RR = 3.20) and autumn (RR = 2.88). This could be a consequence of the seasonality of the digestive process. These findings should be supported by further research. Although not statistically significant, the most cases of carcass condemned by insufficient bleeding were also produced in summer (RR = 1.56); probably due to an increase of fatigued pigs, a consequence of higher temperatures.

Winter was the season with lower percentage of post mortem condemnations (0.0816). Winter was the season with higher percentages only for cases of pleuropneumonia. The result was not statistically significant but this fact could be explained by the seasonally of outbreaks of *Actinobacillus pleuropneumoniae*.

Regarding infectious diseases, only erysipelas and porcine dermatitis nephropathy syndrome were registered after macroscopic inspection. The 49 observed cases of pig erysipelas skin lesions [33] represent a prevalence of 0.0074% (7.36% of the condemned pig carcasses). These percentages were higher than observed by García-Díez and Coelho [5] 0.77%, Vial and Reist [7] 4.8%, or Martínez et al [11] 1.2%. Significantly highest risk of erysipelas was observed in autumn (RR = 5.485) and spring, (2.901) mainly in the months of October and May (Figure 4). Erysipelas is a multifactorial disease [34,35]. The abrupt chances of temperature and the increases of rainfall during these months (Table 1) would be predisposing factors. Erysipelas is an occupational disease [36], so that the risk of infection in workers who encounter sick animals or infected blood and meat makes ante-mortem diagnosis of vital importance. Increased knowledge of environmental factors that may increase the appearance of the disease in this region will make it easy to detect in live animals.

## CONCLUSION

We conclude that the pathologies leading to the condemnation of carcasses in this study can be considered representative of the pathologies that affect the pig population from a region with a high intensive production and Mediterranean climatology because this slaughterhouse receives a lot of animals from many farms of different size from a high intensive pig production zone (Mediterranean region). This knowledge is essential for establishing inspection programs based on risk in slaughterhouses as confirm erysipelas researches in this study. Increased knowledge of environmental factors that may foment the appearance of the diseases in this region facilitated its detection in live animals. In addition, the register of condemnation orders should be improved before communicating them to the referent authorities and included in the food chain information of the animals intended for future sacrifice. Only in this case, official controls can be safely applied in pig slaughterhouses based on risk assessment.

## Figures and Tables

**Table 1:** Average, standard deviation, minimum and maximum value of T^a^ and rainfall per season and month of sacrifice from 2002–2016

Month/season	Average T^a^ (°C)	SD	Mín–Máx T^a^ (°C)	Average rainfall (L/m^2^)	SD	Mín–Max T^a^ (°C)
January	12.51	1.36	11.0–15.8	16.23	18.54	1.4–066.7
February	12.57	1.40	09.9–15.3	13.19	10.00	0.2–035.0
March	14.84	1.07	13.4–16.9	22.60	19.12	0.0–061.8
Winter	13.31	1.67	09.9–16.9	17.34	16.53	0.0–066.7
April	17.25	1.14	15.3–19.7	31.13	24.42	2.0–084.7
May	20.23	1.29	17.1–22.5	24.37	30.86	0.2–111.3
June	24,38	0.96	22.4–26.0	9.83	18.95	0.0–074.2
Spring	20.62	3.16	15.3–26.0	21.78	26.23	0.0–111.3
July	26.99	0.75	25.9–28.8	1,04	1.93	0.0–006.9
August	27.05	0.82	25.7–28.4	9.75	13.4	0.0–043.6
September	24.38	0.83	22.6–25.8	37.22	41.61	0.2–137.2
Summer	26.14	1.48	22.6–28.8	16.00	29.21	0.0–137.2
October	20.97	1.11	19.4–22.8	28.52	33.78	0.0–130.5
November	15.82	1.02	13.8–17.6	31.87	25.68	0.5–104.8
Dececember	12.78	0.90	11.1–14.3	25.04	27.55	0.2–103.0
Autumn	16.52	3.56	11.1–22.8	28.48	28.68	0.0–130.5
Average	19.15	5.47	9.9–28.8	20.90	25.92	0.0–137.2

T^a^, temperature; SD, standard deviation.

**Table 2:** Frequency of condemnations: cause and proportion (%) of total condemnations and total carcasses

Cause	N° condemned carcasses	% of total condemnations	% of total carcasses
Generalized (pyemias)	227	34.08	0.0357
Emaciation	112	16.82	0.0176
Anaemia	71	10.66	0.0111
Erysipelas	49	7.36	0.0074
Pleuropneumonia	39	5.86	0.0061
Insufficient bleeding	29	4.35	0.0045
Arthritis,osteomyelitis	28	4.20	0.0044
PDNS	28	4.20	0.0044
Peritonitis	23	3.45	0.0036
Jaundice	23	3.45	0.0036
Tuberculosis	8	1.20	0.0013
Contamination	7	1.05	0.0011
Pyelonephritis	6	0.90	0.0009
Haemorrhages,edemas	4	0.60	0.0006
Pericarditis	4	0.60	0.0006
Sarcosporidiosis	2	0.30	0.0003
Metritis	1	0.15	0.0002
Cryptosporidiosis	1	0.15	0.0002
Ascaridiasis	1	0.15	0.0002
Melanomas	1	0.15	0.0002
Ptyriasis rosea	1	0.15	0.0002
Odour	1	0.15	0.0002
Total	666	100	0.1046

PDNS, Porcine Dermatitis Nephropathy Syndrome.

**Table 3:** Frequency of condemnations caused by anaemia, proportion (%) of total condemnations and total carcasses

Causes of anaemia	N° condemned carcass by anaemia	% of total anaemias	% of total condemnations	% of total carcasses
Gastric ulcers	34	47.89	5.10	0.0053
Haemorrhagic enteritis	34	47.89	5.10	0.0053
Dysentery	11	29.58	3.15	0.0033
Proliferative	21	15.49	1.65	0.0017
Clostridiosis	2	2.82	0.30	0.0003
Leucosis	3	4.22	0.45	0.0004
Total	71	100	10.66	0.0111

**Table 4:** Mean percentage (%) and effect of season on the rate of condemnation causes

Season	% RR	95%CI	p-value	% RR	95%CI	p-value	% RR	95%CI	p-value
	**Antemortem**			**Postmortem**			**Generalized**		
Autumn	0.0584 1.29	0.54–1.81	0.14	0.1218 1.69*	1.24–2.31	<0.05	0.0306 1.16	0.78–1.71	0.48
Summer	0.0765 1.57*	0.51–2.23	<0.05	0.0998 1.26	0.92–1.72	0.15	0.0377 1.11	0.75–1.65	0.61
Spring	0.0374 0.78	0.26–1.14	0.20	0.1003 1.24	0.92–1.66	0.19	0.0361 1.14	0.76–1.70	0.52
Winter	0.0501 1			0.0816 1			0.0312 1		
	**Emaciation**			**Anaemia**			**Erysipelas**		
Autumn	0.0261 1.74	0.86–3.51	0.13	0.0127 2.88**	1.36–6.07	<0.01	0.0151 5.48**	2.29–13.13	<0.01
Summer	0.0139 1.15	0.54–2.44	0.72	0.0161 3.20**	1.50–6.83	<0.01	0.0008 0.19	0.03–1.48	0.11
Spring	0.0108 0.65	0.29–1.41	0.27	0.0126 2.14	0.98–4.67	0.06	0.0087 2.90*	1.03–8.13	<0.05
Winter	0.0132 1			0.0052 1			0.0029 1		
	**Ins.bleeding**			**PDNS**			**Pleuropneumonia**		
Autumn	0.0039 1.00	0.34–2.90	0.99	0.0041 1.31	0.42–4.11	0.65	0.0064 0.83	0.28–2.41	0.73
Summer	0.0068 1.56	0.57–4.24	0.38	0.0012 0.35	0.07–1.71	0.20	0.0074 1.03	0.42–2.48	0.96
Spring	0.0029 0.58	0.17–2.04	0.40	0.0070 2.18	0.04–6.58	0.17	0.0046 0.70	0.25–1.94	0.49
Winter	0.0046 1			0.0026 1			0.0079 1		
	**Arthritis**			**Peritonitis**			**Jaundice**		
Autumn	0.0085 1.82	0.65–5.09	0.25	0.0050 1.63	0.53–5.00	0.40	0.0037 1.43	0.54–3.77	0.47
Summer	0.0033 0.83	0.26–2.65	0.75	0.0025 0.62	0.19–2.10	0.45	0.0040 1.35	0.51–3.56	0.55
Spring	0.0033 0.79	0.24–2.58	0.69	0.0028 0.88	0.25–3.01	0.83	0.0024 0.77	0.26–2.23	0.63
Winter	0.0040 1			0.0032 1			0.0027 1		

RR, risk ratio; 95% CI, 95% confidence interval;

* p<0,05;

** p<0.01.
